# Distribution of coronary artery calcium in a large European all-comer population referred for cardiac imaging

**DOI:** 10.1016/j.ijcha.2025.101792

**Published:** 2025-09-07

**Authors:** Julia Duelli, Christoph Ryffel, Magdalena Stuetz, Raffael Ghenzi, Marko Gajic, Dimitrios Moysidis, Dominik C. Benz, Aju P. Pazhenkottil, Andreas A. Giannopoulos, Philipp A. Kaufmann, Ronny R. Buechel

**Affiliations:** Department of Nuclear Medicine, Cardiac Imaging, University and University Hospital of Zurich, Raemistrasse 100, CH-8091 Zurich, Switzerland

**Keywords:** Coronary artery calcium scoring, Reference values, Risk stratification, Cardiovascular disease

## Abstract

**Background and aims:**

The coronary artery calcium score (CACS) is a well-established surrogate marker of atherosclerotic plaque burden and is highly valuable for risk stratification. However, contemporary data on the distribution of CACS across age and sex is lacking, particularly for European countries and quantified by multi-slice CT. We assessed predictors of CACS and provide granular age- and sex-specific reference values derived from a real-world clinical population referred for cardiac imaging.

**Methods:**

This single-center, retrospective study examined patients clinically referred for non-invasive cardiac imaging from May 2013 to May 2024. Patients without coronary artery disease, cardiomyopathy, cardiac surgery or intervention, renal or hepatic failure were included. Multiple linear regression was used to identify independent predictors of CACS, and predictor importance was calculated. CACS percentiles were then calculated for both sexes and stratified across nine age groups.

**Results:**

The final population consisted of 18′225 individuals (39.6 % women; 60.8 % symptomatic). Age and sex were the most influential predictors of CACS, accounting for 69 % and 18 % relative predictor importance, respectively. Men exhibited a significantly higher median CACS than women (59 [IQR 1–338] vs. 10 [IQR 0–129], *p* < 0.001) across all age groups.

**Conclusions:**

This study provides contemporary age- and sex-based CAC score reference values, as observed in a large European real-world cohort referred for cardiac imaging. Age and sex exhibit the most relevant impact on expected CAC scores, while other conventional risk factors appear to be less important. Our results enable improved classification regarding the coronary calcium burden.

## Graphical abstract

**Figure:**
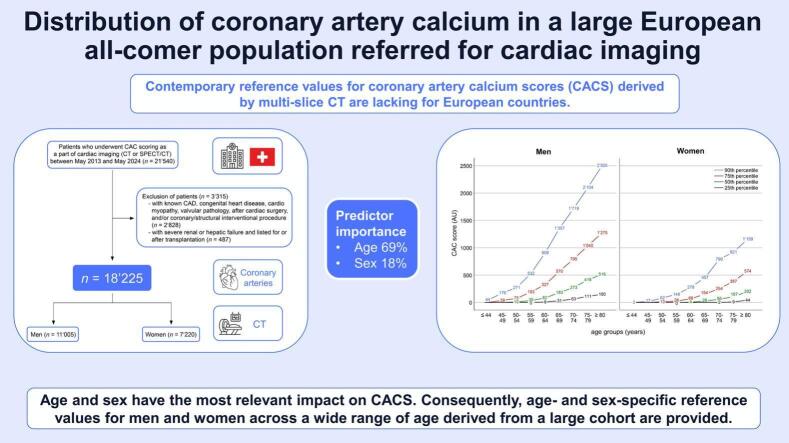

## 1 Introduction

The coronary artery calcium (CAC) score, obtained from non-contrast computed tomography (CT), is a vital clinical tool used in conjunction with traditional risk factors for assessing an individual's risk of future cardiovascular events [1]. Although novel imaging biomarkers such as the pericoronary fat attenuation index are emerging and hold promise for refined risk assessment in patients with suspected or known coronary artery disease (CAD) [2], CAC scoring remains the most widely available and easiest technique for risk stratification at present, whereby the Agatston score is the most widely used method, quantifying the weighted sum of calcification in the coronary arteries with a strictly defined acquisition protocol. The CAC score serves as a direct marker of coronary atherosclerosis and an indirect indicator of CAD, which can be used to decide on preventive drug therapy and lifestyle modifications [[3], [4], [5]].

Coronary artery calcification progresses throughout a lifetime and develops differently in men and women, mandating age- and sex-specific CAC score reference values for accurate risk assessment [6]. However, current reference values are primarily derived from (mostly historical) studies performed in the USA and may not be fully applicable to populations in other regions due to genetic, environmental, and lifestyle differences [[7], [8], [9]]. There are limited studies involving European participants [[10], [11], [12]], and specifically, a notable lack of data originating from European countries classified as low-risk for future cardiovascular events by current European prevention guidelines [13], impeding the ability to provide highly tailored risk assessments for these specific populations. In addition, the reference values are mostly derived from self-referred, asymptomatic participants who do not necessarily represent the patient collective seen in daily routine practice. Finally, given that most historical data are reported from electron beam computed tomography (EBCT), there is an unmet need for contemporary data using state-of-the-art multi-slice detector computed tomography (MDCT) scanners. Although small studies have shown no substantial interscan variability between EBCT and 64-slice CT, the comparability between EBCT and contemporary state-of-the-art MDCT scanners with whole-heart coverage (e.g, 256-slice CT) remains to be elucidated [14,15].

The aim of the present study was to establish contemporary CAC score reference values based on the most important independent predictors for a real-world European population referred for cardiac imaging, with CAC scores obtained using state-of-the-art CT technology. Importantly, the current population consists of all-comers, reflecting a real-world clinical patient collective from daily clinical practice.

## 2 Materials and methods

The local ethics committee approved the study (BASEC-Nr. 2024-01124) and granted an exemption for the need for written informed consent in those patients who did not explicitly refuse to use their personal data for research.

### 2.1 Patient selection

This is a retrospective, single-center study. From our institutional electronic health records, we screened 21′540 patients who underwent CAC scoring on a 256-slice CT scanner (Revolution CT, GE Healthcare, Chicago, IL, USA) as part of clinically indicated non-invasive cardiac imaging (coronary CT angiography or single photon emission computed tomography) between May 2013 and May 2024. Patients with known CAD, congenital heart disease, cardiomyopathy, valvular pathology, those listed for or with a history of transplantation due to severe renal or hepatic failure, a history of cardiac surgery, or coronary intervention were excluded.

### 2.2 Assessment of risk factors and medication

Clinical cardiac symptoms, medication (aspirin, beta-blockers, statins, angiotensin-converting-enzyme inhibitors (ACEI)/angiotensin II receptor blocker (ARB) or nitrates), and cardiovascular risk factors were assessed according to clinical routine before the CT scan. Obesity was defined as a body mass index (BMI) > 30 kg/m^2^ according to patient-reported weight and height. Current or former smoking was defined as current or past use of cigarettes, respectively. Arterial hypertension was defined as treated with antihypertensive medications or as known but untreated hypertension. Diabetes was defined as elevated haemoglobin A1c (HbA1c) above 6.0 % or currently receiving insulin or oral hypoglycaemic medication. Dyslipidaemia was defined as the presence of untreated hypercholesterolaemia. A positive family history of premature CAD was considered present if CAD had occurred in a first-degree relative at ≤65 years and ≤55 years for women and men, respectively.

### 2.3 Imaging protocol and CAC scoring

All patients underwent a CAC scan as per clinical routine: The scan was acquired within one heartbeat using prospective electrocardiogram triggering set at 75 % of the R-R-interval, with the following scan parameters as previously reported: 120 kVp, 200 mA, 256 x 0.625 mm collimation with a z-coverage of 12–16 cm [16]. All datasets were transferred to a workstation (Advantage Workstation AW, GE Healthcare), running a commercially available, semi-automatic software for CAC scoring (SmartScore, GE Healthcare). All pixels with attenuation equal to or above the lower threshold (i.e., ≥130 Hounsfield units) having an area ≥1 mm^2^ are automatically colour-marked, and lesions are manually selected. The software then calculates the CAC score according to the Agatston method [3].

### 2.4 Estimation of clinical likelihood of obstructive CAD and risk

The risk factor-weighted clinical likelihood for obstructive CAD was calculated according to the models by Winther et al. [17], as suggested by the current ESC guidelines for the management of chronic coronary syndromes [18]. Additionally, the 10-year risk of fatal and non-fatal cardiovascular events was estimated according to the SCORE2- and SCORE2-OP risk calculators as provided in the current ESC guidelines on cardiovascular disease prevention [13]. Systolic blood pressures and non-HDL cholesterol levels were imputed according to the patients’ medication and risk factors, whereby elevated systolic blood pressures and/or non-HDL cholesterol were assumed in patients with untreated arterial hypertension and/or untreated dyslipidaemia, respectively.

### 2.5 Statistical analysis

All statistical analyses were performed in SPSS (version 29.0, IBM Corp., Armonk, NY, USA). CAC score percentile ranks were calculated for men and women and stratified across nine age groups with a minimum of 200 subjects per subgroup (i.e. ≤ 44, 45 to 49, 50 to 54, 55 to 59, 60 to 64, 65 to 69, 70 to 74, 75 to 79, and ≥ 80 years) [19]. Age stratification and choice of percentile ranges provided in the tables were selected to ensure comparability with previous studies [8,10]. Quantitative variables are expressed as the mean ± standard deviation (SD) or as median with interquartile range (IQR) if not normally distributed. The data were tested for normal distribution using the Kolmogorov–Smirnov test. Categorical variables are expressed as frequencies or percentages. The chi-square test was used to compare dichotomous variables. The Mann–Whitney *U* test was applied for non-normally distributed variables, and the Jonckheere-Terpstra test for ordered alternatives where the independent variable was of an ordinal type. Additionally, multiple linear regression was applied to identify possible predictors of the CAC score out of the following variables: age, sex, BMI, and cardiovascular risk factors (i.e. current or former smoker, arterial hypertension, diabetes, and a positive family history) and statin medication. As sensitivity analyses revealed that a substantial collinearity between the predictors dyslipidaemia and statin medication was present, with statin use capturing most of the variance attributable to dyslipidaemia, the former was included in the regression model. CAC scores were log-transformed to account for heteroscedasticity. Tests for excluding the presence of multicollinearity were applied. The Durbin-Watson statistical test was used to test for autocorrelation. Standardized coefficients (β), i.e. beta weights, were calculated. Finally, relative predictor importance was estimated using a Random Forest regression model, which was fitted with age, sex, BMI, and cardiovascular risk factors as predictors of log-transformed CAC scores. Predictor importance was quantified by the mean decrease in impurity and normalized to sum to 100 %. To obtain the 95 % confidence intervals (95 % CI), we performed nonparametric bootstrapping with 2′000 resamples and calculated percentile-based intervals from the bootstrap distributions.

A relative predictor importance of ≥10 % was defined as exhibiting a clinically relevant influence. All tests were two-sided, and a *p-value* of < 0.05 was considered statistically significant.

## 3 Results

### 3.1 Study population and baseline characteristics

**Fig. 1:**
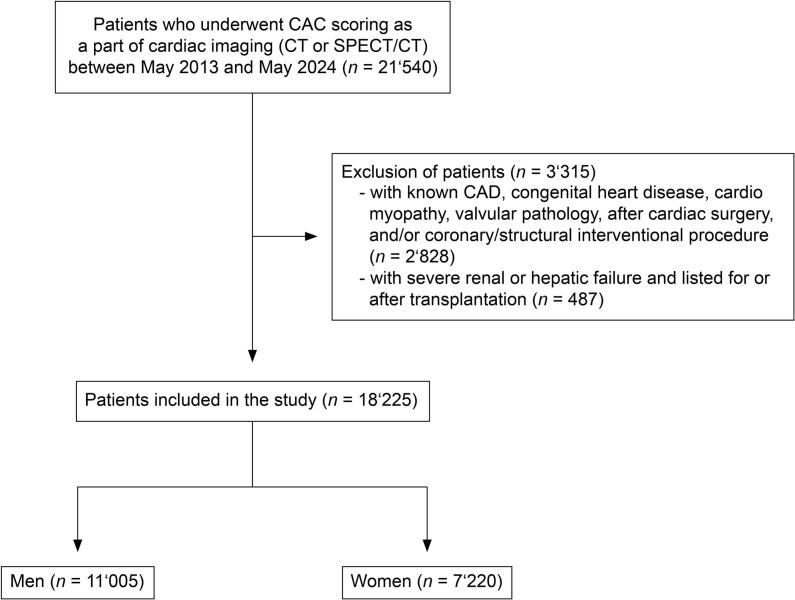
Consolidated Standards of Reporting Trials Diagram of Patient Enrolment.

After exclusion of 3′315 (15.4 %) patients (Fig. 1), the final population consisted of 18′225 individuals (39.6 % women). The median age was 60 (IQR 53–69) years for men and 64 (IQR 56–72) years for women. The clinical characteristics stratified by sex are given in Table 1. The majority of patients (60.8 %) underwent cardiac imaging because of symptoms suspicious for CAD. The remaining patients (39.2 %) were referred due to nonspecific symptoms (e.g., palpitations, syncope, fatigue, etc.) or incidental findings during a check-up or pre-surgical examination (e.g., pathological electrocardiogram or echocardiography, coronary calcifications on chest imaging, etc.) in patients without specific cardiac symptoms.

**Table 1:** Clinical characteristics, risk factors and medication in the overall population and stratified by sex and symptoms.

		All	Men	Women	*p*
	*n*	18′225	11′005 (60.4)	7′220 (39.6)	
Age (yrs)		62 [54 – 70]	60 [53 – 69]	64 [56 – 72]	<0.001
BMI (kg/m^2^)		26.9 [23.5 – 29.4]	26.5 [24.2 – 29.4]	25.4 [22.5 – 29.4]	<0.001

Cardiovascular risk factors					
Obesity		4′013 (22.0)	2′413 (21.9)	1′600 (21.2)	0.709
Current or former smoker		5′267 (28.9)	3′503 (31.8)	1′764 (24.4)	<0.001
Arterial hypertension		7′854 (43.1)	4′759 (43.2)	3′095 (42.9)	0.615
Diabetes		2′122 (11.6)	1′371 (12.5)	751 (10.4)	<0.001
Dyslipidaemia		7′266 (39.9)	4′403 (40.0)	2′863 (39.7)	0.632
Positive family history		4′473 (24.5)	2′492 (22.6)	1′981 (27.4)	<0.001

Medication					
Aspirin		2′898 (15.9)	1′797 (16.3)	1′101 (15.2)	0.510
Beta-blockers		2′739 (15.0)	1′471 (13.4)	1′268 (17.6)	<0.001
Statins		3′808 (20.9)	2′444 (22.2)	1′364 (18.9)	<0.001
ACEI or ARB		4′856 (26.6)	2′966 (27.0)	1′890 (26.2)	0.248
Nitrates		56 (0.4)	32 (0.3)	24 (0.3)	0.619

Estimated clinical likelihood of obstructive CAD					
Very low (≤5%)		4′442 (24.4)	1′787 (16.2)	2′655 (36.8)	<0.001
Low (>5–15 %)		7′002 (38.4)	3′759 (34.2)	3′243 (44.9)	<0.001
Moderate (>15–50 %)		6′594 (36.2)	5′27 (47.9)	1′322 (18.3)	<0.001
High (>50–85 %)		187 (1.0)	187 (1.7)	0 (0.0)	<0.001
Very high (>85 %)		0 (0.0)	0 (0.0)	0 (0.0)	

Estimated 10-year risk of cardiovascular events*					
Low		6′092 (34.1)	2′670 (24.8)	3′422 (48.0)	<0.001
Moderate		9′024 (50.5)	6′203 (57.7)	2′821 (39.6)	<0.001
High		2′757 (15.4)	1′874 (17.4)	883 (12.4)	<0.001

Abbreviation: BMI: body mass index, ACEI = angiotensin-converting enzyme inhibitor, ARB = angiotensin II receptor blocker, yrs = years. Values given are median and IQR in brackets or absolute numbers and percentages in parentheses.

* available for 17′873 patients with age within the range provided by SCORE2 and SCORE2-OP [13].

### 3.2 Predictors of CAC score

**Fig. 2:**
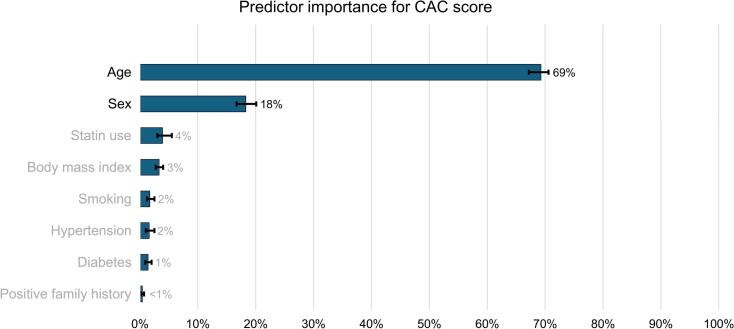
Relative predictor performance for CAC scores. Whiskers depict the 95 % confidence intervals. All variables are independently predicting CAC scores at *p* < 0.001.

Multiple linear regression analysis revealed that the model statistically significantly predicted the CAC score (F(8,18216) = 1162.331, adjusted r^2^ = 0.338, *p* < 0.001). All variables added independently and statistically significantly to the prediction: age (β = 0.499, *p* < 0.001), female sex (β = -0.258, *p* < 0.001), statin use (β = 0.098, *p < 0.001*), current or former smoking (β = 0.079, *p* < 0.001), diabetes (β = 0.062, *p* < 0.001), arterial hypertension (β = 0.060, *p* < 0.001), BMI (β = 0.029, *p < 0.001*), and a positive family history for CAD (β = 0.027, *p* < 0.001). Analysis of relative predictor importance showed that the most important predictors were age (69.3 %, 95 % CI 67.2–70.6 %) and sex (18.3 %, 95 % CI 16.7–20.1 %), while the importance of all other predictors was found to be clinically less relevant (Fig. 2).

### 3.3 Sex- and age-based CAC score percentiles distribution

**Fig. 3:**
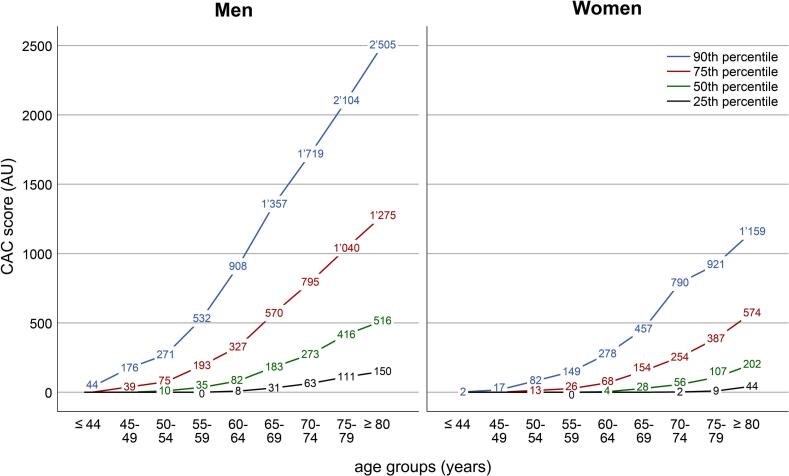
CAC score percentiles for men and women across age groups.

The median CAC score in the overall population was 33 (IQR 0–245, range 0−11′916). Men exhibited a significantly higher median CAC score than women (59 [IQR 1–338] vs. 10 [IQR 0–129], *p* < 0.001) across all age groups. Table 2 provides the observed CAC scores and corresponding percentiles across age groups stratified by sex. Of note, CAC scores differed significantly between men and women across all age groups (*p* < 0.001 for all pairwise comparisons), with men exhibiting higher CAC scores (Fig. 3).

**Table 2:** Distribution of CAC scores by age groups and sex.

		Age groups (yrs)								
All (*n* = 18′225)										
		≤44	45–49	50–54	55–59	60–64	65–69	70–74	75–79	≥80
Percentiles	*n*	980	1′510	2′396	2′821	3′019	2′565	2′170	1′620	1′144
25th		0	0	0	0	0	7	18	38	80
50th		0	0	2	13	35	86	146	224	399
75th		0	16	50	122	217	364	543	710	890
90th		30	118	212	422	680	931	1′226	1′587	1′835

Men (*n* = 11′005)										
		≤44	45–49	50–54	55–59	60–64	65–69	70–74	75–79	≥80
Percentiles	*n*	733	1′015	1′664	1′779	1′836	1′466	1′172	818	522
25th		0	0	0	0	8	31	63	111	150
50th		0	0	10	35	82	183	273	416	516
75th		1	39	75	193	327	570	795	1′040	1′275
90th		44	176	271	532	908	1′357	1′719	2′104	2′505

Women (*n* = 7′220)										
		≤44	45–49	50–54	55–59	60–64	65–69	70–74	75–79	≥80
Percentiles	*n*	247	495	732	1′042	1′183	1′099	998	802	622
25th		0	0	0	0	0	0	2	9	44
50th		0	0	0	0	4	28	56	107	202
75th		0	0	13	26	68	154	254	387	574
90th		2	17	82	149	278	457	790	921	1′159

CAC score values are given as absolute numbers in Agatston Units (AU) for the 25th, 50th, 75th and 90th percentile. Abbreviation: yrs = years.

The results of a subgroup analysis with further stratification into symptomatic versus asymptomatic patients are provided in the supplementary Appendix A, [23], [24], [25], [26], [27], [28], [29], [30], [31]. Notably, asymptomatic men had slightly higher CAC scores than symptomatic men (*p* = 0.037), whereas the difference between symptomatic and asymptomatic women did not reach statistical significance (*p* = 0.092). Finally, an additional subgroup analysis with further stratification into patients with versus without any risk factors revealed higher CAC scores for both women and men with risk factors (*p* < 0.001 for both comparisons). The observed CAC scores and corresponding percentiles are presented in the supplementary Table S2. Of note, however, an explorative multiple linear regression analysis including only age, sex, BMI, and the presence/absence of risk factors (F(4,18220) = 2101.461, adjusted r^2^ = 0.316, *p* < 0.001) revealed that sex and age remained the most important predictors with 77 % and 21 % relative predictor importance.

## 4 Discussion

The current study provides age- and sex-specific reference values for CAC scoring obtained by state-of-the-art CT technology with whole-heart coverage. The cohort studied here represents a real-world patient collective from daily clinical practice within a European low-risk country [13], and the CAC score reference values provided, therefore, can be considered representative for such an all-comer population. Furthermore, our results shed light on the relative predictor importance of potential factors influencing CAC scores and demonstrate that sex and age are the primary drivers of CAC scores, while other factors are less important.

First, it is essential to note that the total number of subjects (*n* = 18′225) and case number per each age group (*n* ≥ 247) in our study are large enough to provide clinically meaningful reference values for both men and women. This stands in contrast to other contemporary European studies, which failed to reach the recommended 200 subjects per age group or could not provide data with sufficient granularity to serve as reference values [12,19,20]. Second, all patients were referred for assessment of suspected CAD, pre-surgical evaluation, or risk stratification. Therefore, our cohort consists of patients representative of daily clinical practice seen in cardiac imaging, including asymptomatic and symptomatic patients. Given the referral setting, however, it is worth noting that our results cannot be necessarily extrapolated to healthy subjects in a screening setting. Finally, the same 256-slice CT scanner, representing a contemporary state-of-the-art CT scanner with whole-heart coverage, was used for all scans, with a consistent acquisition protocol and identical scoring software, which ensures reproducible results for over 10 years.

Sex and age were found to be the most influential predictors of CAC scores, with higher CAC scores observed in men and an increase with age in both sexes. These findings align with previous studies' results, which have repeatedly demonstrated that age, followed by sex, are the strongest and most persistent predictors of CAC score progression [8,21]. Other cardiovascular risk factors, such as diabetes, smoking, and arterial hypertension, exhibited substantially lower relative influence on CAC scores (all < 10 %). However, it remains important to note that our model explained only about 33 % of the variation in CAC scores, suggesting that other unknown predictors, such as genotype-based risk factors or inflammatory markers, may play an important role [2,22]. Future research should explore such additional variables.

The results of this study, specifically an accurate percentile estimation, are important for both an accurate initial assessment of an individual’s CAC burden in relation to a broad population and for the detection and monitoring of overt progression of CAD. In such cases, a deviation from the initially established percentile may provide insights into the effectiveness of preventive measures or the need to intensify them. Furthermore, accurate monitoring of percentiles provides an important indirect contribution to improved individual risk stratification beyond outcome prediction based on absolute CAC values. Finally, yet importantly, accurate percentile estimations can be of significant importance for research. To support decision-making in clinical practice and research, we offer an online calculator for calculating individual percentiles (https://www.cardiacimaging.ch/cacs/).

### 4.1 Comparison to other studies

The supplementary Table S3 provides an overview of relevant studies reporting CAC scores. Of note, the sample sizes of studies performed in Europe are modest at best, rendering comparability difficult. We identified and selected the data published by Hoff et al. from the USA and from the Heinz-Nixdorf Recall (HNR) study by Schmermund et al. from Germany for comparison, as both provide data suitable for comparison, particularly regarding age range and sample size [8,11,19]. The 50th and 75th percentiles of CAC scores for men and women, after adjusting for age groups ≤ 44 years, were visually compared with our own results (Supplementary Fig. S1). Although Germany and the USA are defined as countries with a moderate cardiovascular risk according to the Systematic Coronary Risk Evaluation 2 (SCORE-2) [13], the percentile distribution reveals only moderate differences without substantially differing from the present data. This may seem surprising, given the temporal intervals between data collection among the studies, but also because different CT techniques (i.e. EBCT vs. 256-slice CT) were applied to obtain the CAC scores. This finding corroborates the basic robustness and generalizability of calcium scoring by the Agatston method [2]. However, despite the apparent similarities on a population level, differences in percentile ranking may have clinical implications at the individual level, as prophylactic measures, such as more aggressive treatment of risk factors or preventive medication, may or may not be initiated. The availability of regional and contemporary reference values is, therefore, crucial for accurate risk stratification within the context of the prevailing risk constellations in a given region of the world. Additional future studies with a more regional focus would be highly welcome for a better understanding of the risk distribution among specific populations worldwide.

## 5 Limitations

Beyond the fact that this is a single-center study, which may limit generalizability, our study has other noteworthy limitations. This includes the assessment of cardiovascular risk factors, clinical symptoms indicating a chronic coronary syndrome and medication use. Due to the retrospective nature of this study, we had to rely on the clinically collected and documented information given as per the routine clinical assessment, but outside the context of standardised research protocols. Along the same line, while data on cardiovascular medication was obtained, we lacked any information on the duration of therapy, preventing any meaningful assessments on the effect of medication on CAC scores. Additionally, we had to rely on binary cross-sectional assessment of risk factors, likely preventing us from capturing the continuum of lifetime exposure to risk factors such as dyslipidaemia. Furthermore, despite using multivariable-adjusted models, residual confounding cannot be excluded with certainty and is likely present, given that our model explained only part of the variation in CAC scores. Finally, no outcome data is available for the population studied. Hence, the prognostic value of CAC percentiles derived from our results remains unproven. Nevertheless, the ability to accurately estimate initial and follow-up percentiles improves individual risk stratification beyond absolute CAC values.

## 6 Conclusions

This study provides contemporary age- and sex-based CAC score reference values, as observed in a large European real-world cohort referred for cardiac imaging. In line with previous studies, we found that age and sex have the most relevant impact on expected CAC scores, while other conventional risk factors appear to be less important. Comparable data on this scale were not yet available for Europe. Our results enable improved classification regarding the coronary calcium burden.

## CRediT authorship contribution statement

**Julia Duelli:** Writing – review & editing, Writing – original draft, Visualization, Project administration, Methodology, Investigation, Formal analysis. **Christoph Ryffel:** Writing – review & editing, Investigation. **Magdalena Stuetz:** Writing – review & editing, Investigation, Data curation. **Raffael Ghenzi:** Writing – review & editing, Investigation, Data curation. **Marko Gajic:** Writing – review & editing, Investigation, Data curation. **Dimitrios Moysidis:** Writing – review & editing, Investigation, Data curation. **Dominik C. Benz:** Writing – review & editing. **Aju P. Pazhenkottil:** Writing – review & editing. **Andreas A. Giannopoulos:** Writing – review & editing. **Philipp A. Kaufmann:** Writing – review & editing, Supervision, Resources. **Ronny R. Buechel:** Writing – review & editing, Writing – original draft, Visualization, Validation, Supervision, Software, Resources, Project administration, Methodology, Investigation, Formal analysis, Data curation, Conceptualization.

## Declaration of competing interest

The authors declare that they have no known competing financial interests or personal relationships that could have appeared to influence the work reported in this paper.
