# Supplementary figures and images for: Distribution of coronary artery calcium in a large European all-comer population referred for cardiac imaging
Source: Int J Cardiol Heart Vasc. 2025 Sep 7;61:101792. doi: 10.1016/j.ijcha.2025.101792 (PMC12446614; doi:10.1016/j.ijcha.2025.101792)

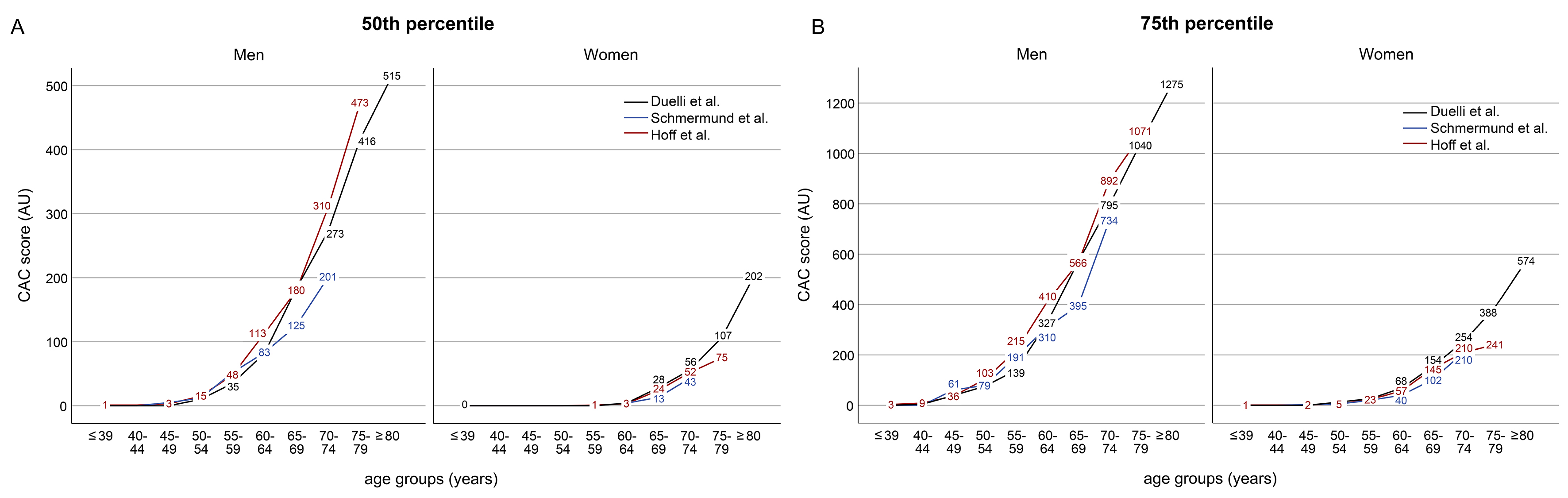

Supplement: Supplementary Figure 1 [file mmc1.jpg]
