# Supplementary material for: Distribution of coronary artery calcium in a large European all-comer population referred for cardiac imaging
Source: Int J Cardiol Heart Vasc. 2025 Sep 7;61:101792. doi: 10.1016/j.ijcha.2025.101792 (PMC12446614; doi:10.1016/j.ijcha.2025.101792)
Supplement: Supplementary Data 2 [file mmc2.docx]

**Table S1** Distribution of CAC scores by age groups and sex for asymptomatic and symptomatic patients

|  |  | | **Age groups (yrs)** | | | | | | | | |
| --- | --- | --- | --- | --- | --- | --- | --- | --- | --- | --- | --- |
| **Asymptomatic men** (*n* = 5’049) | | | | | | | | | | | |
|  |  | ≤44 | | 45-49 | 50-54 | 55-59 | 60-64 | 65-69 | 70-74 | 75-79 | ≥80 |
| **Percentiles** | *n* | 280 | | 464 | 822 | 857 | 858 | 697 | 518 | 346 | 207 |
| 25^th^ |  | 0 | | 0 | 0 | 1 | 9 | 38 | 69 | 103 | 181 |
| 50^th^ |  | 0 | | 0 | 10 | 46 | 87 | 186 | 291 | 381 | 577 |
| 75^th^ |  | 3 | | 35 | 80 | 213 | 325 | 593 | 852 | 1’056 | 1’438 |
| 90^th^ |  | 85 | | 208 | 290 | 571 | 872 | 1’359 | 1’748 | 2’500 | 2’877 |
| **Symptomatic men** (*n* = 5’956) | | | | | | | | | | | |
|  |  | ≤44 | | 45-49 | 50-54 | 55-59 | 60-64 | 65-69 | 70-74 | 75-79 | ≥80 |
| **Percentiles** | *n* | 453 | | 551 | 842 | 922 | 978 | 769 | 654 | 472 | 315 |
| 25^th^ |  | 0 | | 0 | 0 | 0 | 7 | 26 | 56 | 121 | 130 |
| 50^th^ |  | 0 | | 0 | 9 | 30 | 75 | 170 | 260 | 446 | 487 |
| 75^th^ |  | 0 | | 40 | 73 | 182 | 330 | 540 | 758 | 1’021 | 1’169 |
| 90^th^ |  | 31 | | 158 | 240 | 499 | 916 | 1’362 | 1’717 | 1’979 | 2’370 |
| **Asymptomatic women** (*n* = 2’102) | | | | | | | | | | | |
|  |  | ≤44 | | 45-49 | 50-54 | 55-59 | 60-64 | 65-69 | 70-74 | 75-79 | ≥80 |
| **Percentiles** | *n* | 82 | | 139 | 244 | 349 | 371 | 328 | 263 | 189 | 137 |
| 25^th^ |  | 0 | | 0 | 0 | 0 | 0 | 0 | 0 | 9 | 82 |
| 50^th^ |  | 0 | | 0 | 0 | 0 | 6 | 34 | 50 | 98 | 259 |
| 75^th^ |  | 0 | | 0 | 13 | 41 | 72 | 162 | 243 | 342 | 663 |
| 90^th^ |  | 23 | | 22 | 84 | 172 | 286 | 525 | 774 | 1’172 | 1’690 |
| **Symptomatic women** (*n* = 5’118) | | | | | | | | | | | |
|  |  | ≤44 | | 45-49 | 50-54 | 55-59 | 60-64 | 65-69 | 70-74 | 75-79 | ≥80 |
| **Percentiles** | *n* | 165 | | 356 | 488 | 693 | 812 | 771 | 735 | 613 | 485 |
| 25^th^ |  | 0 | | 0 | 0 | 0 | 0 | 0 | 2 | 9 | 36 |
| 50^th^ |  | 0 | | 0 | 0 | 0 | 3 | 25 | 60 | 108 | 163 |
| 75^th^ |  | 0 | | 0 | 13 | 23 | 66 | 148 | 264 | 403 | 542 |
| 90^th^ |  | 1 | | 17 | 82 | 125 | 276 | 426 | 810 | 847 | 1’114 |

CAC score values are given as absolute numbers in Agatston Units (AU) for 25^th^, 50^th^, 75^th^ and 90^th^ percentile for asymptomatic and symptomatic men and women. Abbreviations: yrs = years

| **No risk factors, men** (*n* = 2’183) | | | | | | | | | | |
| --- | --- | --- | --- | --- | --- | --- | --- | --- | --- | --- |
|  |  | ≤44 | 45-49 | 50-54 | 55-59 | 60-64 | 65-69 | 70-74 | 75-79 | ≥80 |
| **Percentiles** | *n* | 158 | 212 | 364 | 363 | 344 | 269 | 200 | 158 | 115 |
| 25^th^ |  | 0 | 0 | 0 | 0 | 0 | 15 | 29 | 75 | 116 |
| 50^th^ |  | 0 | 0 | 1 | 11 | 27 | 120 | 176 | 348 | 346 |
| 75^th^ |  | 0 | 7 | 38 | 102 | 180 | 404 | 506 | 858 | 1’010 |
| 90^th^ |  | 8 | 72 | 152 | 297 | 556 | 993 | 1’144 | 2’386 | 2’036 |
| **≥ 1** **risk factors, men** (*n* = 8’822) | | | | | | | | | | |
|  |  | ≤44 | 45-49 | 50-54 | 55-59 | 60-64 | 65-69 | 70-74 | 75-79 | ≥80 |
| **Percentiles** | *n* | 575 | 803 | 1’300 | 1’416 | 1’492 | 1’197 | 972 | 660 | 407 |
| 25^th^ |  | 0 | 0 | 0 | 2 | 13 | 36 | 70 | 119 | 166 |
| 50^th^ |  | 0 | 1 | 13 | 50 | 99 | 202 | 293 | 444 | 577 |
| 75^th^ |  | 2 | 46 | 88 | 228 | 376 | 613 | 845 | 1’061 | 1’405 |
| 90^th^ |  | 62 | 214 | 313 | 612 | 968 | 1’497 | 1’886 | 2’048 | 2’659 |
| **No risk factors, women** (*n* = 1’486) | | | | | | | | | | |
|  |  | ≤44 | 45-49 | 50-54 | 55-59 | 60-64 | 65-69 | 70-74 | 75-79 | ≥80 |
| **Percentiles** | *n* | 74 | 133 | 161 | 199 | 202 | 202 | 209 | 170 | 136 |
| 25^th^ |  | 0 | 0 | 0 | 0 | 0 | 0 | 0 | 3 | 29 |
| 50^th^ |  | 0 | 0 | 0 | 0 | 0 | 7 | 27 | 55 | 209 |
| 75^th^ |  | 0 | 0 | 0 | 5 | 23 | 113 | 157 | 280 | 525 |
| 90^th^ |  | 0 | 6 | 39 | 41 | 161 | 263 | 568 | 756 | 1’079 |
| **≥ 1** **risk factors, women** (*n* = 5’734) | | | | | | | | | | |
|  |  | ≤44 | 45-49 | 50-54 | 55-59 | 60-64 | 65-69 | 70-74 | 75-79 | ≥80 |
| **Percentiles** | *n* | 173 | 362 | 571 | 843 | 981 | 897 | 789 | 632 | 486 |
| 25^th^ |  | 0 | 0 | 0 | 0 | 0 | 0 | 3 | 16 | 48 |
| 50^th^ |  | 0 | 0 | 0 | 0 | 6 | 30 | 65 | 116 | 199 |
| 75^th^ |  | 0 | 0 | 17 | 37 | 85 | 164 | 291 | 419 | 590 |
| 90^th^ |  | 5 | 21 | 109 | 171 | 293 | 492 | 825 | 957 | 1’293 |

**Table S2** Distribution of CAC scores by age groups and sex for patients with versus without cardiovascular risk factors.

CAC score values are given as absolute numbers in Agatston Units (AU) for 25^th^, 50^th^, 75^th^ and 90^th^ percentile for men and women without risk factors or ≥ 1 risk factors. Abbreviations: yrs = years

| **Author** | **Year** | **Country** | **Total**  **n** | **Men**  **n** | **Women**  **n** | **%** ♀ | **Age** | **Min. Age** | **Max. Age** | | **Symptoms** | **CAD** | **Scanner** | **Referred by** | **Details** | |  |
| --- | --- | --- | --- | --- | --- | --- | --- | --- | --- | --- | --- | --- | --- | --- | --- | --- | --- |
| Mitchell [7] | 2001 | USA | 18’785 | 12’169 | 6616 | 35 | n.a. | ♂ > 35  ♀ > 40 | > 74 | | n.a. | no | EBCT | self or physician | no comparison due to small sample size >74y | |  |
| **Hoff [8]** | **2001** | **USA** | **35’246** | **25’251** | **5775** | **16** | **51** | **30** | **90** | | **No** | **no** | **EBCT** | **self** |  | |  |
| Schmermund [10] | 2002 | Germany | 2030 | 1515 | 515 | 34 | 56 ± 10 | n.a. | n.a. | | No | no | 4S-CT | Heinz Nixdorf Recall study | no comparison due to small sample size per age group | |  |
| Nasir [23] | 2004 | USA | 12’936 | 8720 | 4216 | 33 | 53 | n.a. | n.a. | | no | no | EBCT | physician | no comparison due to use of calcium volume score | |  |
| Dakik [24] | 2005 | Lebanon | 1154 | 938 | 216 | 19 | 52 | 26 | 80 | | no | no | EBCT | self | no comparison due to small number per age group, self-payer | |  |
| McClelland [9] | 2006 | USA | 6110 | 1195 | 1308 | 53 | 62 | n.a. | n.a. | | no | no | different | MESA cohort | no comparison due to exclusion of diabetics and using different scanners at 6 centers. Data of white participants are given. | |  |
| **Schmermund [11]** | **2006** | **Germany** | **4275** | **2027** | **2248** | **53** | **59 ± 8** | **45** | **74** | | **n.a.** | **no** | **EBCT** | **Hein Nixdorf Recall study** |  | |  |
| Budoff [25] | 2007 | USA | 25’253 | 13’659 | 11’594 | 46 | 56 ± 11 | n.a | n.a. | | no | no | EBCT | physician | registry, long follow up, comparison to 10’377 patient series for near-term to long-term survival | |  |
| Hoffmann [26] | 2008 | USA | 3238 | 1652 | 1586 | 48 | 53 | ♂ ≥ 35  ♀ ≥ 40 | n.a. | no | | no | 8S-CT | Framingham study | | no comparison due to too young participants | |
| Gudmundsson [12] | 2012 | Iceland | 4266 | 1593 | 2673 | 62 | 76 ± 5 | n.a. | n.a. | | n.a. | yes | 4S-CT | cohort of Reykjavik study | no comparison due to other age groups, small sample sizes, and older study population. Data given without participants after coronary event (28%) | |  |
| Pereira [27] | 2016 | Brasil | 2135 | 1021 | 1114 | 56 | 50 | 35 | 74 | | no | no | 64S-CT | ELSA-Brasil | no comparison due to other age groups and exclusion of diabetics, data of white participants are given | |  |
| Jang [28] | 2016 | Korea | 31’545 | 26’174 | 5371 | 21 | 54 ± 8.3 | 20 | ≥ 80 | | no | no | 64S-CT | self/voluntarily | no comparison due to other ethnic group | |  |
| Blaha [29] | 2017 | USA | 54’678 | 44’633 | 22’003 | 33 | 54 ± 11 | n.a | n.a | | no | no | different | physician CAC Consortium | CAC Consortium, no comparison due to lack of data for percentiles | |  |
| Wang [6] | 2019 | USA | 70’320 | 46’378 | 23’942 | 34 | 54 ± 10 | < 40 | > 75 | | no | no | 93% EBCT 7% MDCT | self or physician,  part of CAC Consortium | no comparison due to number of plaques no CAC scores | |  |
| Garcia [20] | 2020 | Spain | 220 | 124 | 96 | 54 | 58 | 35 | 75 | | no | no | 256S-CT | randomly selected population | no comparison due small sample size per age group | |  |
| Javaid [30] | 2022 | USA | 19’725 | n.a | n.a | n.a | n.a | 30 | 45 | | no | no | different |  | pooled analysis, no comparison due to too younger participants | |  |
| Kılıçkap [31] | 2024 | Turkey | 3941 | 2250 | 1691 | 43 | 52 ± 11 | n.a. | n.a. | | n.a. | yes | 512S-CT |  | no comparison due to small sample size per age group | |  |
| **Duelli** |  | **Switzerland** | **18’225** | **11’005** | **7220** | **40** | **62 [54-70]** | **16** | **96** | | **y** | **no** | **256S-CT** | **physician** |  | |  |

**Table S3** Overview of relevant studies providing distributions of coronary artery calcium scores

Studies marked in bold were considered for comparison. Abbreviations: ♀ = women; ♂= men; AMI = acute myocardial infarction; CAD = coronary artery disease; EBCT = electron beam computer tomography; MDCT = multi-detector computer tomography; n.a. = not available; Year = year of publication. Values given are mean ± SD, absolute numbers and percentages in parentheses or median and IQR in brackets.
